# Information content in genome-wide scans: concordance between patterns of genetic differentiation and linkage mapping associations

**DOI:** 10.1186/1471-2164-12-65

**Published:** 2011-01-26

**Authors:** Pamela Wiener, Mohammad A Edriss, John L Williams, David Waddington, Andrew Law, John A Woolliams, Beatriz Gutiérrez-Gil

**Affiliations:** 1 The Roslin Institute and R(D)SVS, University of Edinburgh, Roslin, Midlothian EH25 9PS, UK; 2 Department of Animal Science, College of Agriculture, Isfahan University of Technology, Isfahan 8415683111, Iran; 3 Parco Tecnologico Padano, Via Einstein, Polo Universitario, Lodi 26900, Italy; 4 Departamento de Producción Animal, Facultad de Veterinaria, Universidad de León, 24071 León, Spain

## Abstract

**Background:**

Scanning the genome with high density SNP markers has become a standard approach for identifying regions of the genome showing substantial between-population genetic differentiation, and thus evidence of diversifying selection. Such regions may contain genes of large phenotypic effect. However, few studies have attempted to address the power or efficacy of such an approach.

**Results:**

In this study, the patterns of allele frequency differences between two cattle breeds based on the Bovine HapMap study were compared with statistical evidence for QTL based on a linkage mapping study of an experimental population formed by a cross between the same breeds. Concordance between the two datasets was seen for chromosomes carrying QTL with strong statistical support, such as BTA5 and BTA18, which carry genes associated with coat color. For these chromosomes, there was a correspondence between the strength of the QTL signal along the chromosome and the degree of genetic differentiation between breeds. However, such an association was not seen in a broader comparison that also included chromosomes carrying QTL with lower significance levels. In addition, other chromosomal regions with substantial QTL effects did not include markers showing extreme between-breed genetic differentiation. Furthermore, the overall consistency between the two studies was weak, with low genome-wide correlation between the statistical values obtained in the linkage mapping study and between-breed genetic differentiation from the HapMap study.

**Conclusions:**

These results suggest that genomic diversity scans are capable of detecting regions associated with qualitative traits but may be limited in their power to detect regions associated with quantitative phenotypic differences between populations, which may depend on the marker resolution of the study and the level of LD in the populations under investigation.

## Background

With the development of dense genome-wide marker panels for many species, it is becoming common to use these markers to characterize genetic diversity across the genome. Such genomic scans are designed to identify regions where selection has acted and which therefore, may contain genes of large phenotypic effect. The rationale is that, even without phenotypic information, one can use patterns of genetic variation to highlight genomic regions under selection. However, the power and reliability of these studies has not been assessed because usually there is no independent data set against which the results can be compared. The current study was designed to address this point by examining the concordance between a genomic diversity dataset comprising genome-wide SNP data from two cattle breeds and a set of linkage mapping results from a study in an F2/Backcross population bred from founders of these same breeds.

Using these datasets, an overall concordance between level of genetic differentiation and linkage mapping signal was evaluated. In addition, the more specific question to be addressed is whether regions with large allele frequency differences between the breeds are more likely to contain genes controlling phenotypes that differ between the breeds than regions with small allele frequency differences. However, because not all of the traits that might distinguish the breeds can be measured, the question was addressed in the converse direction in this study, i.e. whether regions of the genome where QTL have been identified are characterized by larger SNP allele frequency differences compared with other genomic regions.

## Methods

### Interval mapping experiment

#### Source population

A three-generation Charolais x Holstein cattle herd was bred from seven founder Charolais sires, which were mated with Holstein cows to produce 137 F1 animals. From these, a total of 501 second generation cross-bred animals were produced, 315 were F2 individuals from crossing eight F1 sires with F1 cows, and 186 were reciprocal backcrosses of the F1 animals with the founder breeds (88 Charolais backcrosses, CB1, and 98 Holstein backcrosses, HB1). The second generation animals were measured for a wide variety of traits [1-5] (results for dairy traits have not yet been published). For the analysis presented here, a subset of traits related to growth, dairy and meat production and coat colour were selected, as these traits were expected to have been under the strongest divergent selection in the two founder breeds. Specific traits were selected that were representative of these trait groups but were not strongly correlated with each other. These included size and growth rate, coat color, carcass conformation characteristics, detailed carcass measurements, meat quality traits (as assessed by a trained taste panel), chemical composition of meat and milk, milk yield and udder characteristics (Additional File 1, Table S1). Other trait groups were not included (e.g. behavioral and immunological traits). All work in this study involving the use of animals was designed under guidance of and carried out under a United Kingdom Government Home Office animal experimentation license, under the supervision of the local Home Office inspector, and all procedures were formally inspected annually.

#### Microsatellite marker data

A panel of 165 microsatellite markers were genotyped in the population; 139 of these were selected at random from across the autosomes and the other 26 were added later in regions on nine chromosomes where QTL were detected following initial analysis [4]. Linkage maps were constructed using CRIMAP 2.4 [6]. The maps obtained were compared with the latest published version of the bovine linkage map [7] and found to be consistent.

Microsatellite markers used to derive the linkage maps were then mapped against the Baylor bovine genome assembly Btau_4.0 [8] in order to derive genomic sequence coordinates, either by extracting pre-computed locations from Ensembl (http://www.ensembl.org/Bos_taurus) (88 markers) or by megaBLAST [9] sequence similarity searches using flanking sequence (64 markers). The sequence positions of the remaining 13 markers, for which a definitive megaBLAST-based location could not be identified within the expected genomic location, were assigned estimated positions based on their proximity to flanking markers on the genetic linkage map.

#### Interval mapping analysis

The QTL analysis was performed using the QTL Express software [10], which implements the linear regression method of Knott and Haley [11] assuming the founder lines to be fixed for alternative alleles at the QTL loci. A single QTL model analysis with additive and dominance effects was fitted at intervals of 100,000 basepairs along each chromosome according to the maps described above. Fixed effects considered in the linear model and the numbers of phenotypes analyzed are given in Additional file 1, Table S1. Fixed effects included genetic composition (F2, CB1, and HB1) for all traits as well as sex, birth cohort, dairy cohort, taste panel grouping, feeding regime and background coat colour, as detailed in Additional file 1, Table S1. The F-ratio for each combination of trait and chromosomal position was recorded. The maximum of F-ratios (F_max_) over all traits at each chromosomal position was used as the test statistic for the interval mapping results.

### Bovine HapMap study

#### Genomic diversity data

Genome-wide SNP data was obtained from the Bovine HapMap Consortium [12] and is subsequently referred to as HapMap. Details of the HapMap samples, SNP markers, genotypes and quality control have been described previously [12]. Briefly, DNA samples for individuals from *Bos taurus *breeds (as unrelated as possible, based on pedigree), plus sire-dam-offspring trios for data quality assessment, were collected and genotyped for 33,851 biallelic SNPs distributed across the genome (encompassing 3.2 Gb for Assembly Btau_4.0). The majority of SNPs were discovered by comparing shotgun genome sequence reads for cows from six breeds (Angus, Brahman, Holstein, Jersey, Limousin and Norwegian Red) to the Hereford reference genome sequence. SNP density was fairly uniform across the genome except that higher densities of markers were positioned on three chromosomes (BTA6, 14 and 25: average: ~27 per Mbp compared to ~10 per Mbp for the other 26 autosomes).

Only the genotype data for 49 Holstein and 20 Charolais individuals included in the HapMap project was considered (excluding calves that formed part of family trios and one Charolais that was missing a majority of genotypes). Loci with low minor allele frequencies, high error rates, evidence of deviation from Hardy-Weinberg Equilibrium or excessive missing data were removed from the data set (as in the HapMap analysis, [12]), as were loci that could not be positioned on Assembly Btau_4.0 or were found on the X chromosome. This left 31,312 SNPs covering the autosomes. SNP positions along the genome were based on Assembly Btau_4.0.

#### Measures of marker differentiation

To calculate genetic differentiation from the HapMap data, the following protocol was followed. The frequency of one allele was calculated for each SNP for each of the two breeds. Two statistics were then used to characterize the differences between the two breeds. First the absolute value of the difference in allele frequencies between the Holstein and Charolais breed groups (*δ*) was calculated at each SNP position (equivalent to *δ*_*c *_for two alleles [13]). Secondly, estimates of pairwise F_ST _(*θ*) were calculated for each SNP [14,15], adjusting for different sample sizes as in Ref. [16]. If F_ST _estimates were negative, they were set to 0 [16]. The dataset was restricted to those markers for which F_ST _was defined (i.e. the average allele frequency across the two breeds was not 0 or 1). Sequence positions for each marker were rounded to the nearest 100,000 bp and an average value of *δ *(or F_ST_) was calculated for all markers with the same rounded position. Two smoothed statistics, *MA_δ *and *MA_ *F_ST_, were calculated as moving averages of 11 *δ *or F_ST _values, respectively, centered on each rounded position, i.e. including that position and its 10 flanking positions (excluding the first and last 5 positions of the chromosome).

### Data Analysis

Correlation coefficients were calculated between F_max _values from interval mapping and each of the four measures of SNP breed differences from the HapMap study (*δ*, F_ST_, *MA_δ *and *MA_ *F_ST_) over the entire genome and for each chromosome. Then chromosomal positions were grouped according to whether their F_max _value was above (highF) or below (lowF) a given threshold. Three groupings were defined using the approximate cut-offs for chromosome-wise and genome-wide significance previously calculated by permutation testing for this data (chromosome-wise, 0.05, F = 5; chromosome-wise, 0.01, F = 7; genome-wide, 0.01, F = 10; [1-4]). For each of these three thresholds, the mean *δ *and F_ST _values (and their respective moving averages) were calculated for the highF and lowF groups on each chromosome. Numbers of chromosomes for which any F_max _values exceeded the thresholds decreased with threshold values (27 chromosomes for F = 5, 13 chromosomes for F = 7 and 6 chromosomes for F = 10). Wilcoxon signed rank tests were used to test whether the mean values of *δ *and F_ST _(and their respective moving averages) for each chromosome differed for the highF and lowF groups, for each of the three F-thresholds. A one-tailed test was applied, i.e. the alternative hypothesis was that the highF group had greater *δ *or F_ST _values than the lowF group.

## Results

**Figure 1:**
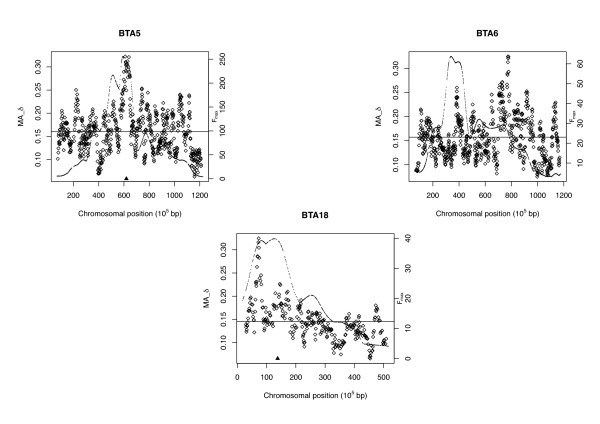
**Patterns of genetic differentiation and linkage mapping results for chromosomes with large QTL **. Patterns of the moving average of allele frequency differences between Holstein and Charolais cattle (*MA_δ*, represented by diamonds) and the maximum F-ratio of the linkage mapping study with Holstein and Charolais founders (F_max_, represented by the curve) across BTA5, 6 and 18. The horizontal lines show the average values of *MA_δ *for each chromosome. For BTA5, the black triangle on the x-axis indicates the position of the *SILV *gene. For BTA18, the black triangle indicates the position of the *MC1R *gene.

The genome-wide correlation between F_ST _and *δ *was high (0.8764) and results for both measures were generally similar. Thus, results are presented only for *δ *(and *MA_δ*) except where qualitative differences were seen between results for *δ *and F_ST_. Visual inspection did not show a consistent trend between F_max _and *δ *across the genome, although such a trend was seen for some chromosomes. Figure 1 shows F_max _and *MA_δ *across the chromosomes with the largest QTL in the study, on BTA5, 6 and 18 (all chromosomes are shown in Additional file 2, Figure S1). The genome-wide correlation coefficient between F_max _and *δ *was 0.0603 and that between F_max _and *MA_δ *was 0.1672. The correlations varied considerably across chromosomes (Table 1), with half of the chromosomes (14/29) showing negative correlations between F_max _and *δ *(and *MA_δ*). BTA5, 6 and 18 had positive correlations although the correlation coefficient was low for BTA6. For the chromosomes with a positive correlation between F_max _and *δ*, correlations were consistently higher between F_max _and *MA_δ *than between F_max _and *δ *(this was also true for F_ST _except for BTA6 and BTA26, for which the correlations were very low). The highest chromosome-wide positive correlations between F_max _and *δ *were (in descending order) for BTA28, 18, 14, 5, 2 and 7 (for the correlations between F_max _and *MA_δ*, the order of BTA28 and BTA18 and that of BTA8 and BTA2 were switched but otherwise the same).

**Table 1:** Summary of F_max_, δ, F_ST _and correlations across autosomes.

Chrom*	Number **of F**_**max**_ values	**Max^#^F**_**ST**_	**Max^#^δ**	**Max^#^F**_**max**_	Correlation between **F**_**max **_**& F**_**ST**_	Correlation between **F**_**max **_**&** ***MA*_ F**_**ST**_	Correlation between **F**_**max **_**& δ**	Correlation between **F**_**max **_**&*MA*_δ**
1	866	0.6149	0.6617	7.0582	-0.0490	-0.1188	-0.0330	-0.0806
2	818	0.5478	0.6389	6.7646	0.1372	0.4355	0.1150	0.3657
3	635	0.5268	0.6015	8.0034	-0.0134	-0.0447	-0.0240	-0.0659
4	635	0.7376	0.6287	6.9333	0.0730	0.2046	0.0445	0.1412
5	660	0.8641	0.7954	255.5152	0.1795	0.4460	0.1660	0.4209
6	721	0.8024	0.8036	63.6723	0.0132	0.0131	0.0342	0.0749
7	519	0.7822	0.7971	7.7939	0.1052	0.2860	0.1028	0.2755
8	593	0.7285	0.7903	5.4648	0.1506	0.3676	0.1199	0.2970
9	447	0.4608	0.5493	5.3908	-0.1054	-0.3073	-0.1056	-0.3037
10	556	0.6959	0.5794	11.3568	-0.0892	-0.2758	-0.0679	-0.2230
11	507	0.5892	0.5034	8.8217	0.0280	0.0983	0.0242	0.0880
12	483	0.4831	0.5136	11.0992	0.0126	0.0239	-0.0237	-0.0847
13	269	0.6742	0.6698	6.8665	0.0053	0.0467	0.0188	0.0770
14	398	0.5125	0.6051	4.0904	0.1636	0.3959	0.1898	0.4449
15	402	0.4853	0.5276	6.3632	0.0485	0.1335	0.0448	0.1353
16	320	0.6282	0.5255	8.7356	0.0371	0.0977	0.0818	0.2140
17	410	0.5530	0.5538	5.2488	-0.0548	-0.1871	-0.0950	-0.2959
18	305	0.6597	0.6103	39.8582	0.2797	0.6698	0.2360	0.6506
19	288	0.4334	0.5522	6.7943	-0.0321	-0.0631	-0.0293	-0.0691
20	379	0.5081	0.6032	6.3753	-0.0043	-0.0147	0.0063	0.0137
21	295	0.5278	0.5618	5.6594	-0.0854	-0.2444	-0.0792	-0.2104
22	340	0.6280	0.6876	11.7822	-0.0882	-0.2496	-0.1624	-0.3923
23	286	0.3849	0.4909	7.8903	-0.0436	-0.1912	-0.0627	-0.2376
24	266	0.6615	0.6156	6.7555	-0.0332	-0.1192	-0.0646	-0.2141
25	144	0.3342	0.4643	5.7339	-0.0943	-0.2887	-0.1508	-0.4656
26	221	0.6427	0.7126	3.8836	0.0169	0.0080	0.0497	0.0877
27	236	0.4251	0.5181	6.1233	-0.0867	-0.1931	-0.0389	-0.0256
28	156	0.4355	0.5076	5.9923	0.2218	0.6162	0.2378	0.6462
29	270	0.6615	0.6253	9.5407	-0.0021	0.0095	-0.0180	-0.0251

*Chrom = Chromosome, ^#^Max = Maximum

To test whether SNP ascertainment bias, and in particular the fact that the majority of SNPs were detected in Holsteins, may explain the lack of concordance between the interval mapping and SNP frequency, the effect of breed-of-origin on the value of *δ *was examined. This statistic was significantly higher (p < 0.001) for the SNPs detected in Holsteins (*δ *= 0.1565) than that detected in all other breeds (0.1372). The correlation coefficient between *δ *and F_max _values was recalculated as described above, for the 14,132 segregating SNPs that were detected in breeds other than Holsteins. For that subset of data, the overall correlation coefficient between F_max _and *δ *was 0.0427, lower than that calculated for all SNPs, thus the inclusion of the Holstein-derived SNPs did not reduce the overall correlation between F_max _and *δ*. However, for the non-Holstein SNP data, fewer chromosomes (13/29) had negative correlations between F_max _and *δ *(12/29 for *MA_δ) *than was found considering all SNPs (14/29).

**Figure 2:**
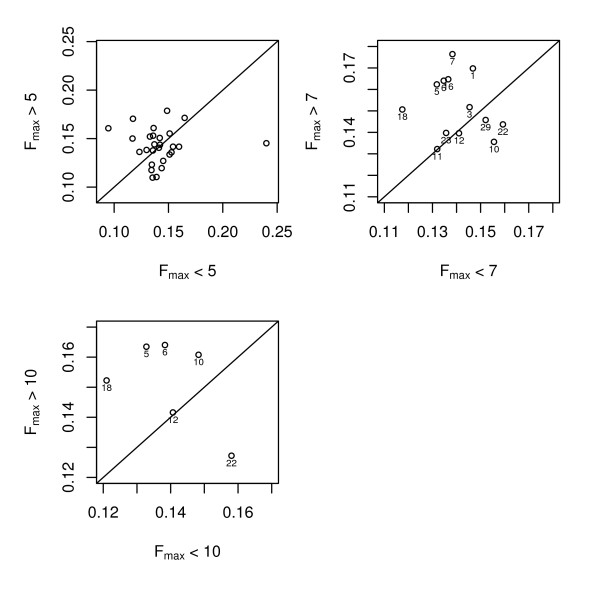
**Genetic differentiation for positions with significant versus non-significant linkage mapping results for three significance thresholds**. Mean moving average of allele frequency differences (*MA_δ*) values across chromosomal positions above and below linkage mapping maximum F-ratio (F_max_) thresholds of 5, 7, and 10. Each circle represents an individual chromosome; only chromosomes for which there were F_max _values exceeding the relevant threshold are included.

The comparison of *δ *and *MA_δ *distributions above and below F_max _thresholds revealed little difference between regions with high and low F_max _values for the lowest F_max _threshold (F = 5), but there were differences for the higher thresholds (F = 7 and F = 10). Wilcoxon signed rank tests revealed significant differences in the mean *δ *values between highF and lowF positions for F_max _thresholds of both 7 and 10 (p < 0.05), but not for the threshold of 5. The same results were seen for comparisons of mean *MA_δ *values between highF and lowF positions (for F_ST _and *MA_ *F_ST_, only tests with threshold F = 10 were significant). These results are presented graphically in plots of the chromosome-wide mean *MA_δ *values for highF versus those for lowF positions (Figure 2). For F = 5, slightly more than half of the chromosomes (15/27) had greater mean *MA_δ *values for highF than lowF groups. For the cut-off of F = 7, nine out of 13 chromosomes had greater mean *MA_δ *values for highF than lowF groups. For the F = 10 cut-off, five out of the six chromosomes for which there were F_max _values greater than 10 had greater mean *MA_δ *values for highF than lowF groups. These five chromosomes included those with the highest F_max _values in this study (255.52, 63.67 and 39.86 for BTA5, 6 and 18, respectively), as well as BTA10 and 12 for which the highest F_max _values were between 10 and 12 (BTA22, for which the mean *MA_δ *value was greater for lowF than highF groups, also had its highest F_max _value in this range).

With regard to extreme values of the genetic differentiation measures across the genome: the top 1% of the *δ *distribution (values > 0.4981) covered 27 of the 29 autosomes. Of the 124 loci in the top 1% of the *δ *distribution, the greatest number of markers were found on BTA1 (9 positions), 5 (13 positions), 6 (10 positions) and 8 (9 positions). When positions were ranked by *MA_δ *values rather than by *δ *(Additional file 3, Table S2), the top 1% (> 0.2622) were present on 18 of the 29 autosomes, with the majority on BTA5 (20 positions), 6 (15 positions), 13 (15 positions) and 20 (13 positions). The most extreme values of *MA_δ *(top 0.1%) were found on BTA5 (5 positions, 61-64 Mbp), BTA6 (5 positions, 77-78 Mbp), and BTA18 (2 positions, 7.2-7.3 Mbp). The highest *δ*, *MA_δ *and *MA_ *F_ST _values were found at 77.4 Mbp on BTA6. The highest F_ST _value (and third highest *δ *value) was found at 63.6 Mbp on BTA5.

## Discussion

The objective of the analysis reported here was to assess if there was consistency between the pattern of allele frequency differences between two cattle breeds and the pattern of statistical evidence for QTL identified in a cross-bred population of the same breeds. The motivation for this was to test whether the current trend to look for signatures of selection in data from high density genome scans could detect the genomic regions controlling phenotypic traits identified by traditional linkage mapping techniques. The results suggest that genomic differentiation patterns can pick up very large, qualitative phenotypic effects, but may have limitations as predictors of genomic regions associated with smaller, quantitative phenotypic differences.

In the comparison of genomic data from two cattle breeds carried out in the present study, allelic differentiation was amplified in regions where the largest genetic effects were detected by QTL mapping (BTA5 and 18), however, overall, there was low correlation between the test statistics from the two analyses. The BTA5 and 18 QTL were associated with Mendelian loci influencing coat colour: the Charolais-derived allele in a region of BTA5 (close to or at the *SILV *gene, 61.9 Mbp) causes complete dilution of coat pigmentation and gives this breed its characteristic white coat [3,17] and the BTA18 QTL is associated with the *MC1R *locus (13.8 Mbp), which is the main regulator of the switch between red and black coat color pigments [18] and associated with black and red coat color in cattle, where the black allele is dominant [19,20]. Charolais carry the red allele at *MC1R*, although it is not visible in the coat because of the dilution effect, whereas black and white Holsteins are predominantly homozygous for the black allele, with a low frequency of heterozygotes [3,21]. The correlations between F_max _and *MA_δ *were 0.421 on BTA5 and 0.651 on BTA18, and as seen in Figure 1, the QTL peaks overlap with peaks in the *MA_δ *graphs. Furthermore, the positions of the known pigmentation genes are very close to the peak *MA_δ *values, particularly for the *SILV *gene on BTA5. However, there was only a weak positive correlation on BTA6 (0.075), where the third highest F-ratio in the linkage mapping study was found. The QTL corresponding to the high F-ratio was associated with birth weight and carcass-related traits [4] and was coincident with QTL for similar traits reported in other studies [22,23]. As seen in Figure 1, there is concordance between this QTL peak and a region of high *MA_δ *although this was not in the top 1% of the distribution. The chromosome-wide correlation on BTA6 was reduced, however, because there are other regions further downstream with even higher values of *MA_δ *but where there is only weak evidence of QTLs, for bone weight and average feed intake. The regions of high *MA_δ *may be associated with traits that were not measured in the linkage mapping study or the linkage mapping study may not have been powerful enough to detect associations in this region. In the 2-Mbp region centered at 77.4 Mbp (the peak *δ *and *MA_δ *position), there are no annotated genes in the bovine genome, although there is homology to some expressed genes in the human genome (http://genome.ucsc.edu). The *KIT *gene, which is associated with the level of white spotting in Holstein cattle and other mammals [24], is located at 72.8 Mbp on this chromosome, nearly 5 Mbp downstream of the *δ *and *MA_δ *peak. While the level of spotting was not analyzed in the linkage mapping population, variation in this trait was observed in the F2/Backcross generation.

Interpretation of correlation coefficients calculated across the genome is made difficult because not all traits that differ between the breeds were measured in the interval mapping study. Indeed some loci under divergent selection in these two breeds may not have phenotypically obvious effects and will not have been analysed. However, this problem is avoided by the analysis where differentiation levels were compared at positions with low F-ratios versus high F-ratios. Where there is a positive association between the two analyses, there should be higher allele frequency differences for the high F-ratio group than the low F-ratio group, even if there are other QTL that were not detected. For the most extreme comparison of F_max _> 10 versus F_max _< 10, there was a clear distinction in mean *δ *values between the lowF and highF groups; 5 of the 6 chromosomes with F_max _values > 10 had greater mean *δ *values for positions with high F-values. The exception was BTA22, for which the mean *δ *(and *MA_δ*) value was higher for the lowF group. There were several QTL on this chromosome for carcass traits, but the *MA_δ *graph did not follow the QTL pattern. While the difference between the highF and lowF groups for the F_max _= 7 threshold was also statistically significant, the effect was not as pronounced as for the F_max _= 10 threshold; for 4 out of 13 chromosomes, *MA_δ *was greater in the lowF than the highF group.

### Efficacy and power of differentiation-based "selection mapping"

While approaches based on allelic differentiation are currently being used for QTL mapping in some model species e.g. in Drosophila, Arabidopsis and maize [25-27], the general applicability of these methods is not yet clear. Theoretical predictions of allele frequency differences at markers linked to a locus under selection suggest that even for a locus under strong selection, the allele frequency differences between two selected lines will fall off rapidly with distance between the markers and the selected locus [28]. A similar effect should hold for multiple populations that are divergent for the same traits, as measured by F_ST _or related statistics. This has been demonstrated in recent empirical studies: e.g. Sutter *et al. *[29] looked at the population structure for 22 large and small dog breeds using dense markers near the *IGF*1 locus, a gene that is associated with body size, a trait which has been under strong selection in the development of dog breeds. In this region, they found a very narrow peak (< 0.2 Mbp) in extreme values of F_ST _(their Figure two). In contrast, Akey *et al. *[30] identified a 1-Mbp region including several markers with high F_ST _values between Shar-Pei and Dachshund dogs (their Figure three) in a region containing a strong candidate locus for skin wrinkling (a characteristic of the Shar-Pei breed). The extensive region of inflated F_ST _in this case compared to the narrower region surrounding *IGF*1 may reflect the more limited region of LD shared by several breeds compared to a broader LD region that may be shared by two breeds. The power of an approach based on allelic differentiation for identification of genes of large effect will depend, in part, on the levels of linkage disequilibrium in the genome. Further studies are required to quantify this relationship and to determine the marker density required to detect loci associated with selected traits in a given population.

### Limitations of the current study

Factors in addition to LD levels may have influenced the results of the present study, for example, the interval mapping data was based on small numbers of individuals measured for some of the phenotypic traits (especially dairy-related traits). This may have increased the rates of both false positive and false negative linkage associations. Furthermore, the density of markers in the linkage study was relatively low such that QTL positions were not very precise and in many cases 95% confidence intervals covered most of the chromosome. However, marker density was enhanced in regions where QTL were detected in preliminary analysis [4], which increased the precision of positioning the QTL with largest effects and therefore should have increased the correlation between allele frequency difference and F_max _for these regions (e.g. BTA6). However, this was not observed in the analysis. In addition, performing interval mapping using genome sequence positions may also have influenced the scale of the F_max _values as there is not a one-to-one correspondence between the linkage map and the genome sequence due, in part, to variation in recombination rates across the genome. This should not, however, have had a substantial impact on the results pertaining to the moving average measures, which were spatially averaged. Finally, although the linkage mapping experiment was designed to detect QTL fixed for alternative alleles in the founder breeds, it may still have picked up large effects for which the QTL was segregating within breeds. In that case, allelic differentiation between breeds would be reduced compared to the case where alternative alleles were fixed in the two breeds.

In addition to the inherent limitations of the individual datasets, there may be problems in combining them: the QTL mapping study used British Charolais and Holstein animals as founders for the experimental crossbred population, while the HapMap SNP diversity study sampled North American Charolais and Holsteins. This should not have been a problem for the Holstein breed, which is very similar worldwide due to large-scale use of artificial insemination in the global dairy industry and the worldwide predominance of North American sire lines. However, there may be genetic differences between UK and North American Charolais due to genetic drift or different selection pressures in Europe and North America. Such differences are most likely to occur in regions associated with quantitative traits rather than highly-visible, categorical traits like coat colour which are key for breed identification.

### High genetic differentiation signal

In addition to the high *MA_δ *values on BTA5, 6 and 18 discussed above, there were extreme values (> 0.3) on BTA13 (11.3 Mbp) and BTA20 (28.5-28.6 Mbp). The BTA13 region could not be associated with a gene known to be involved in any of the traits considered or with QTL for these traits from this study or others. On BTA20, a QTL for growth was detected slightly upstream of the F_max _peak (Additional file 2, Figure S1). Furthermore, the growth hormone receptor (*GHR*) gene, located at 33.9 Mbp on this chromosome, is associated with growth in cattle [31,32] as well as milk yield and composition traits [33]. As in the case with *KIT *on BTA6, however, this gene is several Mbp downstream of the *MA_δ *peak.

Several of the regions with high levels of genetic differentiation were detected in previous studies to identify signatures of selection in cattle. Seven of the 13 regions of high F_ST _reported by Flori *et al. *[34] in three dairy breeds (their Table two) were within 2 Mbp of the top 1% *MA_δ *positions reported in the current study. These included regions of BTA3, 4, 5, 6, 18 (near *MC1R*), 20 (near *GHR*) and 26. In a study of allele frequency differences between Holstein and Angus (another beef breed) cattle [35], of the 15 significant (*p *< 0.001) sliding window average differences (their Table two), three were within 2 Mbp of the top 1% *MA_δ *positions identified in the current study: on BTA14 (two positions) and BTA20, within 5 Mbp of GHR. There was less concordance between data reported here and results from Barendse *et al. *[36], which reported markers with high F_ST _values for both the original HapMap dataset (19 beef and dairy breeds) and a set of 20 beef and dairy breeds from Australia (their Additional File two). Only 4 out of the 95 significant markers identified by Barendse *et al. *[36] were within 2 Mbp of peak regions reported in the current study. These were found on BTA1, 7, 12 and 13. None of the seven regions with high F_ST _reported in the original HapMap study [12] (their Table one) overlapped with regions identified in the current study. However, in a separate analysis of the HapMap data [37], 11 out of 78 regions showing differences in allele frequencies between three or more dairy breeds and the complete dataset (see their Table S2) were located within 2 Mbp of peak regions in the current study.  These covered seven chromosomes and included the region of BTA6 near the *KIT* gene.  The correspondence between the current study and those of Flori *et al*. [34] and Stella *et al*. [37], which focused on dairy breeds, may provide some insight into the development of cattle breeds. It suggests that the major selective pressures have not been specifically for "meat" or "dairy" traits, but rather for more obvious characteristics like coat color or pattern and overall size. This is likely to be in part due to the relative length of time in which selection has been applied to these appearance-related traits as compared to less visible traits related to meat and dairy production, some of which may have been under strong selection only over the last few decades. In addition, color and appearance traits are generally under the control of one or relatively few genes (see the Online Mendelian Inheritance in Animals (OMIA) database, http://www.ncbi.nlm.nih.gov/sites/entrez?db=omia) whereas many meat and dairy traits are quantitative traits and thus under the control of multiple genes, which may fix more slowly than single genes [38]. It is likely that if the current trend of specialization in some cattle breeds (e.g. Holstein) continues, the level of differentiation at loci associated with production traits will also increase.

## Conclusions

The study reported here describes a direct comparison of a linkage mapping study with a genetic differentiation scan for the identification of genomic regions associated with population/breed differences. Results from this study indicate that while the genome-wide analysis of genetic diversity does detect regions associated with large, qualitative phenotypic effects (such as coat color), this approach may not have sufficient power to detect smaller, quantitative effects, depending on the marker resolution used in the study and the levels of linkage disequilibrium in the populations under investigation.

## Authors' contributions

PW planned the study, performed some of the data analyses and drafted the manuscript, MAE wrote and executed scripts to extract and manipulate the data, positioned the markers used in the linkage mapping study on the genome sequence, and performed some of the data analyses, JLW conceived and managed the study from which the linkage mapping results derived, was part of the Bovine HapMap Consortium and participated in writing the manuscript, DW advised on and assisted with the statistical analyses, AL wrote and executed scripts to extract and manipulate the data, JAW advised on the statistical analyses and was part of the Bovine HapMap Consortium, and BGG provided the linkage mapping data, assisted with positioning the markers on the genome sequence and helped prepare the manuscript. All authors approved the manuscript.

## Supplementary Material

Additional file 1 Table S1: List of traits analyzed from F2/Backcross linkage mapping experiment. Click here for file

Additional file 2 **Figure S1: Patterns of the moving average of allele frequency differences between Holstein and Charolais cattle (*MA_δ*, represented by diamonds) and the maximum F-ratio of the linkage mapping study with Holstein and Charolais founders (F**_**max**_**, represented by the curve) across the bovine autosomal genome (BTA1 - BTA29).** Click here for file

Additional file 3 Table S2: Top 1% MA_δ values, sorted by chromosome. Click here for file
