# Supplementary material for: Information content in genome-wide scans: concordance between patterns of genetic differentiation and linkage mapping associations
Source: BMC Genomics. 2011 Jan 26;12:65. doi: 10.1186/1471-2164-12-65 (PMC3041744; doi:10.1186/1471-2164-12-65)
Supplement: Additional file 1 — Table S1: List of traits analyzed from F2/Backcross linkage mapping experiment. [file 1471-2164-12-65-S1.DOC]

Table S1. List of traits analyzed from F2/Backcross linkage mapping experiment.

| **Trait** | **Fixed effects included in model** | **Sample size** |
| --- | --- | --- |
| **Birth and growth traits** |  |  |
| Birth Weight (kg) (males and females) | year of birth^1^, sex | 463 |
| Pre-weaning Growth Rate (kg/day) (males) | year of birth^1^, feeding treatment^2^ | 250 |
| Post-weaning Growth Rate (kg/day) (males) | year of birth^1^, feeding treatment^2^ | 249 |
| Average Feed Intake (females) | year of birth^1^, dairy cohort^3^ | 210 |
| **Slaughter traits (males)** |  |  |
| Subcutaneous fat estimate (%) | year of birth^1^ | 235 |
| Marbling score (1-7 scale) | year of birth^1^ | 235 |
| Conformation score (1-15 scale) | year of birth^1^ | 235 |
| Fat score (12-point scale) | year of birth^1^ | 235 |
| **Commercial dissection traits (males)*** |  |  |
| Total weight of trimmed, commercially dissected joints (bone and fat removed) (dg) | year of birth^1^ | 235 |
| Total weight of bone trim from commercially dissected joints (dg) | year of birth^1^ | 235 |
| **Full-tissue dissection traits (males)*** |  |  |
| Lean weight of forerib primal (dg) | year of birth^1^ | 235 |
| Intermuscular fat weight of forerib primal (dg) | year of birth^1^ | 235 |
| Vertebral weight of forerib primal (dg) | year of birth^1^ | 235 |
| Non-vertebral bone weight of forerib primal (dg) | year of birth^1^ | 235 |
| Lean weight of leg primal (dg) | year of birth^1^ | 235 |
| Intermuscular fat weight of leg primal (dg) | year of birth^1^ | 235 |
| Bone weight of leg primal (dg) | year of birth^1^ | 235 |
| **Taste panel traits (males)** |  |  |
| Texture (roast) | date of taste panel^4^ | 235 |
| Juiciness (roast) | date of taste panel^4^ | 235 |
| Texture (grilled) | date of taste panel^4^ | 235 |
| Juiciness (grilled) | date of taste panel^4^ | 235 |
| **Meat quality traits (males)** |  |  |
| Intramuscular fat | year of birth^1^ | 235 |
| Haem pigment concentration (mg/g) | year of birth^1^ | 235 |
| Moisture content (%) | year of birth^1^ | 235 |
| Yield force (kgf) | year of birth^1^ | 235 |
| **Coat color traits (males and females)** |  |  |
| light red^§^ | -- | 481 |
| black^§^ | -- | 481 |
| dilution (degree of pigment in all animals)^§§^ | color background^5^ | 481 |

| **Milk composition traits (females)** |  |  |
| --- | --- | --- |
| total casein (g/100g milk) **^¥^** | year of birth^1^, dairy cohort^3^ | 137 |
| kappa casein (% total caseins) **^¥^** | year of birth^1^, dairy cohort^3^ | 137 |
| alpha s1 casein (% total caseins) **^¥^** | year of birth^1^, dairy cohort^3^ | 137 |
| alpha s2 (% total caseins) **^¥^** | year of birth^1^, dairy cohort^3^ | 137 |
| beta casein (% total caseins) **^¥^** | year of birth^1^, dairy cohort^3^ | 137 |
| total whey protein (g/100g milk) **^¥^** | year of birth^1^, dairy cohort^3^ | 137 |
| albumin & lactoferrin (% of total whey) **^¥^** | year of birth^1^, dairy cohort^3^ | 137 |
| beta-lactobglobulin (% of total whey) **^¥^** | year of birth^1^, dairy cohort^3^ | 137 |
| total_fat (g/100g milk) **^¥^** | year of birth^1^, dairy cohort^3^ | 137 |
| monounsaturated fat (% total fat) **^¥^** | year of birth^1^, dairy cohort^3^ | 137 |
| polyunsaturated fat (% total fat) **^¥^** | year of birth^1^, dairy cohort^3^ | 137 |
| average protein % | year of birth^1^, dairy cohort^3^ | 210 |
| average fat % | year of birth^1^, dairy cohort^3^ | 210 |
| **Milk yield (females)** |  |  |
| total milk yield (l) | year of birth^1^, dairy cohort^3^ | 212 |
| average daily milk yield (l/day) | year of birth^1^, dairy cohort^3^ | 212 |
| length of lactation (days) | year of birth^1^, dairy cohort^3^ | 212 |
| **Udder traits (females)** |  |  |
| udder volume (l) | year of birth^1^, dairy cohort^3^ | 138 |
| Area of right-hind cistern (cm^2^) | year of birth^1^, dairy cohort^3^ | 138 |
| Area of right-front cistern (cm^2^) | year of birth^1^, dairy cohort^3^ | 138 |
| **Health (females)** |  |  |
| average somatic cell count (cells/ml milk) | year of birth^1^, dairy cohort^3^ | 210 |

1. year of birth: 1998-2001

2. feeding treatment: F2 and CB1 bulls were suckled by their dams until ~6 months, however, HB1 bulls were removed from their dams at birth. Where possible, they were fostered onto other cows, but otherwise, they were raised on milk replacement and weaned at 4 weeks to solid rations [4].

3. dairy cohort: groups of cows that were put through lactation trial together (1-5)

4. date of taste panel: 12 dates on which sensory assessment was conducted [2]

5. color background: red or black [3]

* See Ref. [4] for detailed description of commercial and full-tissue dissection

§ binary traits: 1 = expression of indicated color category (light red or black), 0 = lack of expression; [3]

§§ degree of pigment = 1 (no pigment), 2 (intermediate pigment), 3 (dark pigment); [3]

¥ detailed milk composition traits were measured at both early- and mid-lactation (days 7 and 93); the measurement with greatest evidence of QTL was used for the analysis
