# Supplementary material for: Information content in genome-wide scans: concordance between patterns of genetic differentiation and linkage mapping associations
Source: BMC Genomics. 2011 Jan 26;12:65. doi: 10.1186/1471-2164-12-65 (PMC3041744; doi:10.1186/1471-2164-12-65)
Supplement: Additional file 2 — Figure S1: Patterns of the moving average of allele frequency differences between Holstein and Charolais cattle (MA_δ, represented by diamonds) and the maximum F-ratio of the linkage mapping study with Holstein and Charolais founders (Fmax, represented by the curve) across the bovine autosomal genome (BTA1 - BTA29). [file 1471-2164-12-65-S2.PDF]

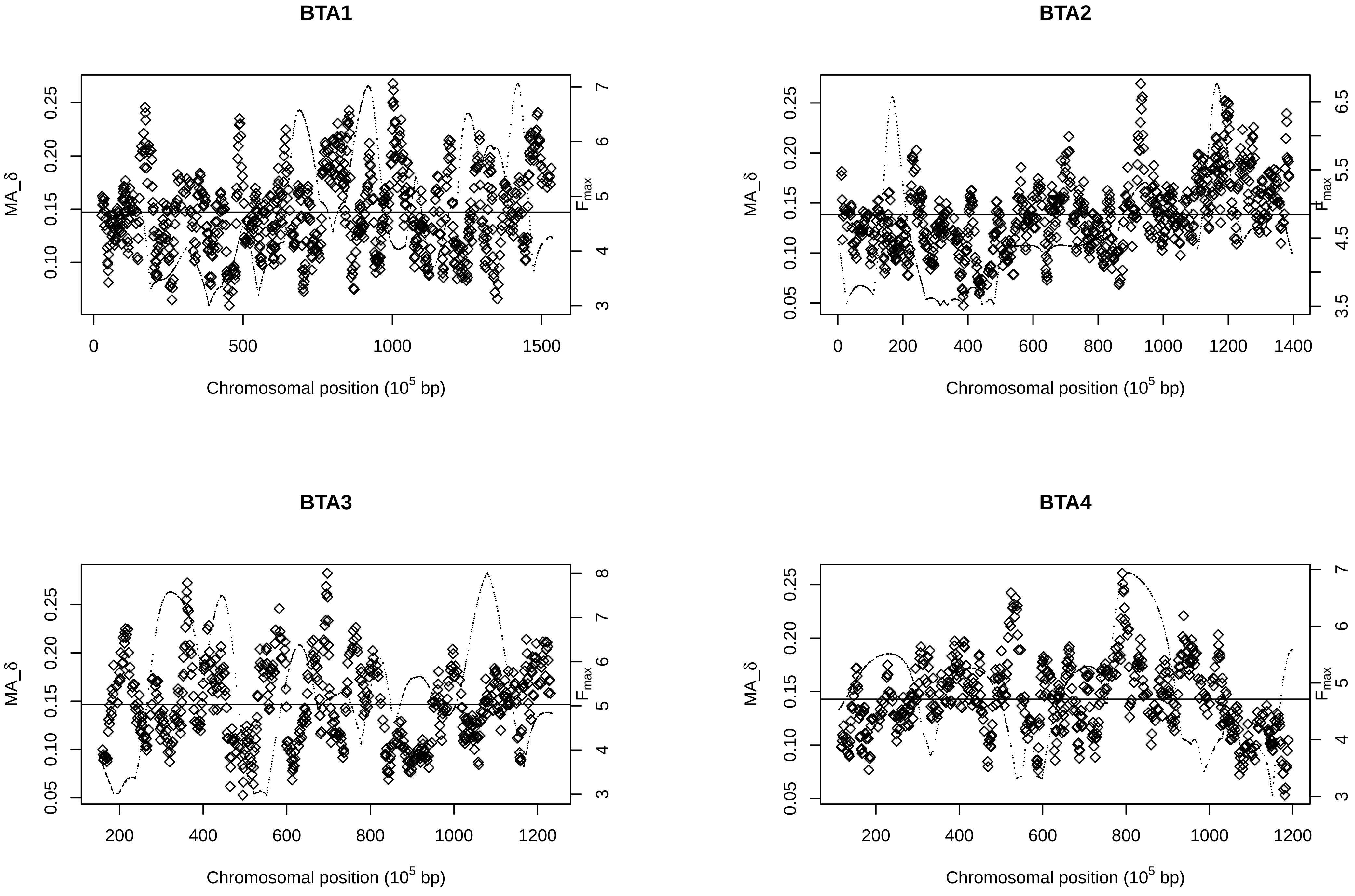
BTA1
BTA2
BTA3
BTA4
MA_δ
F max
Chromosomal position (10^5 bp)
0.05 0.10 0.15 0.20 0.25
0 500 1000 1500
0 200 400 600 800 1000 1200 1400
200 400 600 800 1000 1200
3 4 5 6 7
3.5 4.5 5.5 6.5
3 4 5 6 7 8

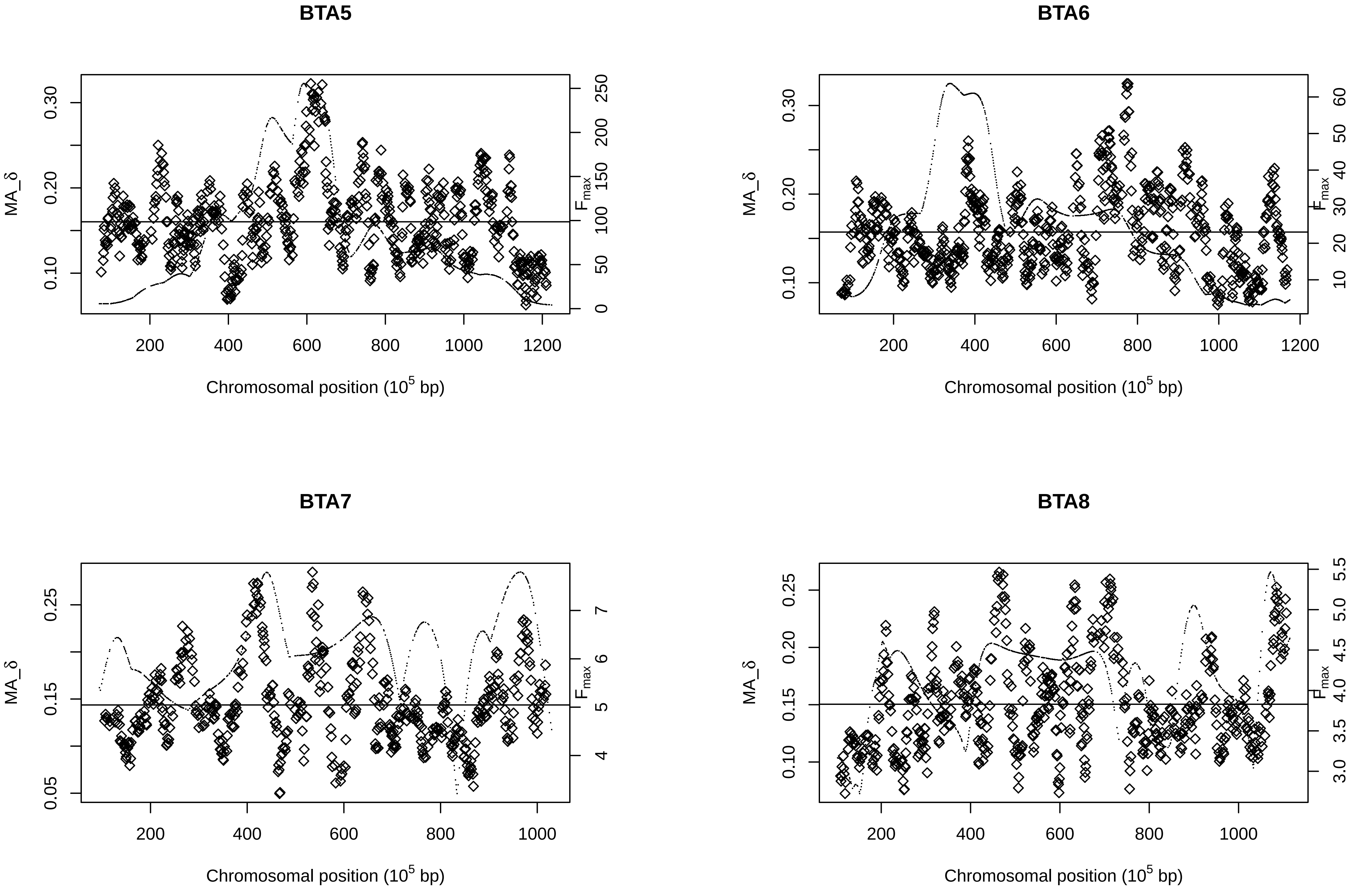
BTA5
BTA6
BTA7
BTA8
MA_δ
F max
Chromosomal position (10^5 bp)
200
400
600
800
1000
1200

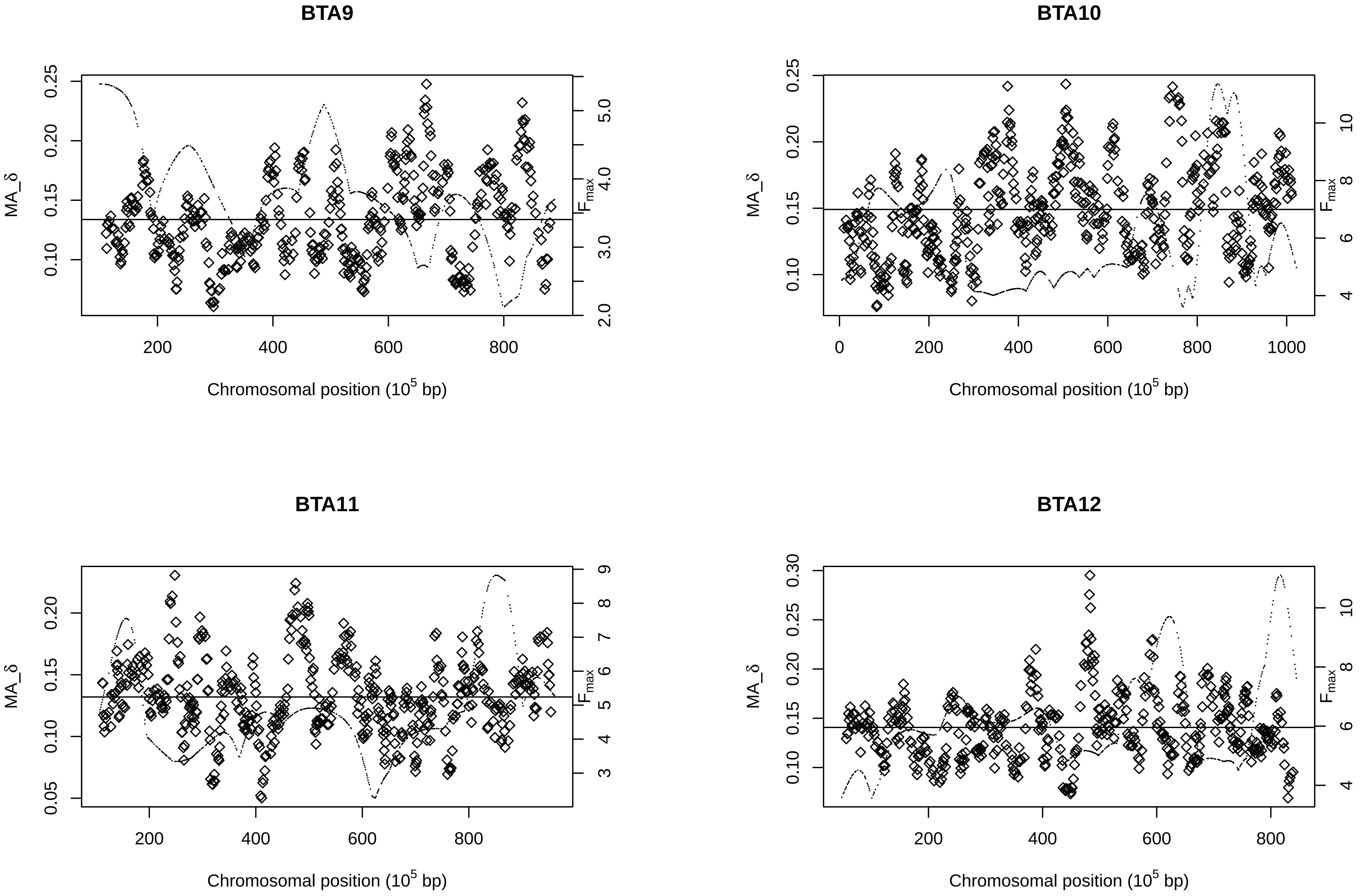
BTA9
BTA10
BTA11
BTA12
MA_δ
F_{max}
Chromosomal position (10^5 bp)

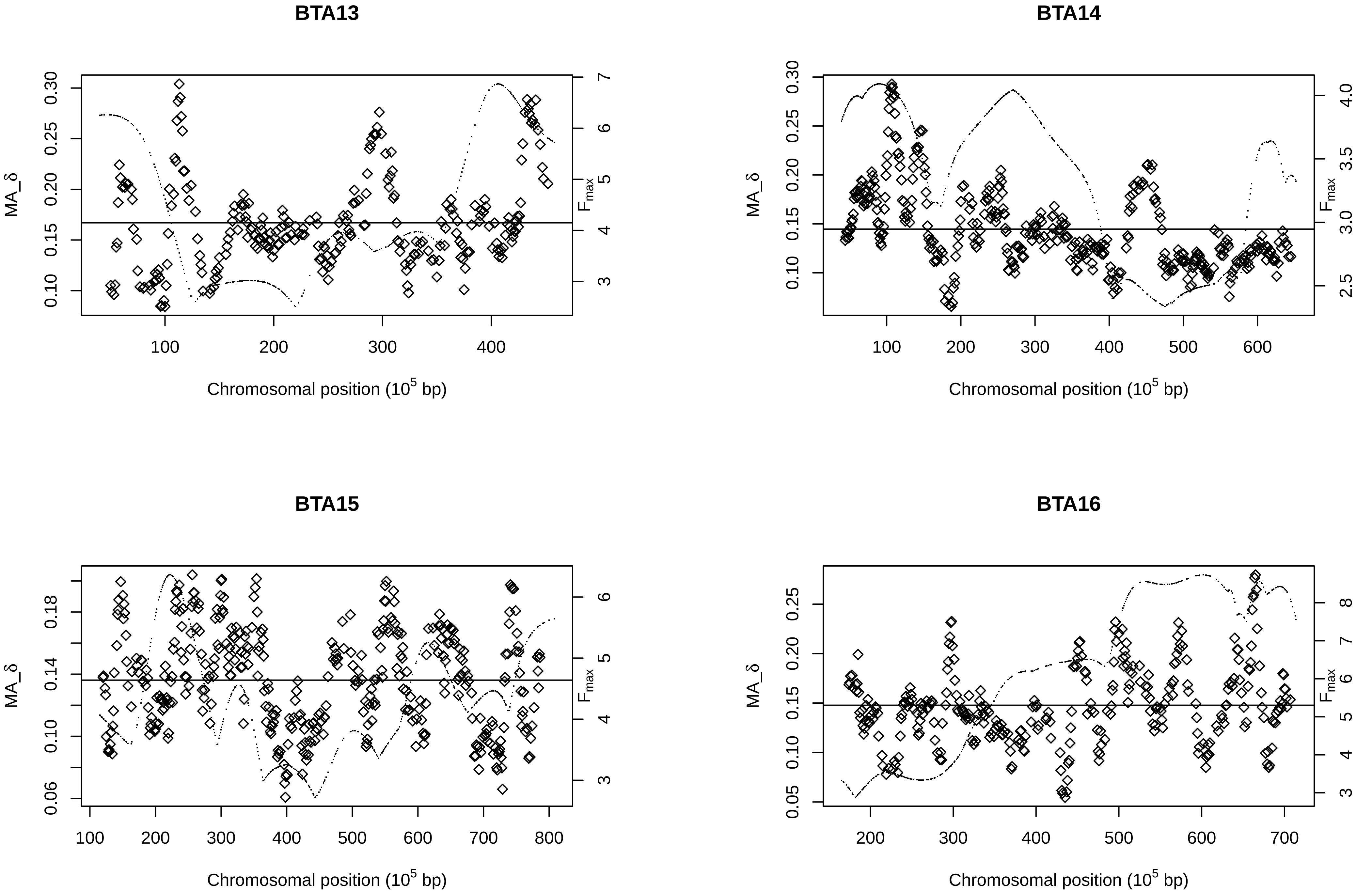
BTA13
BTA14
BTA15
BTA16
MA_δ
F_{max}
Chromosomal position (10^5 bp)
0.10 0.15 0.20 0.25 0.30
3 4 5 6 7
100 200 300 400
2.5 3.0 3.5 4.0
100 200 300 400 500 600
0.06 0.10 0.14 0.18
3 4 5 6
100 200 300 400 500 600 700 800
0.05 0.10 0.15 0.20 0.25
3 4 5 6 7 8
200 300 400 500 600 700

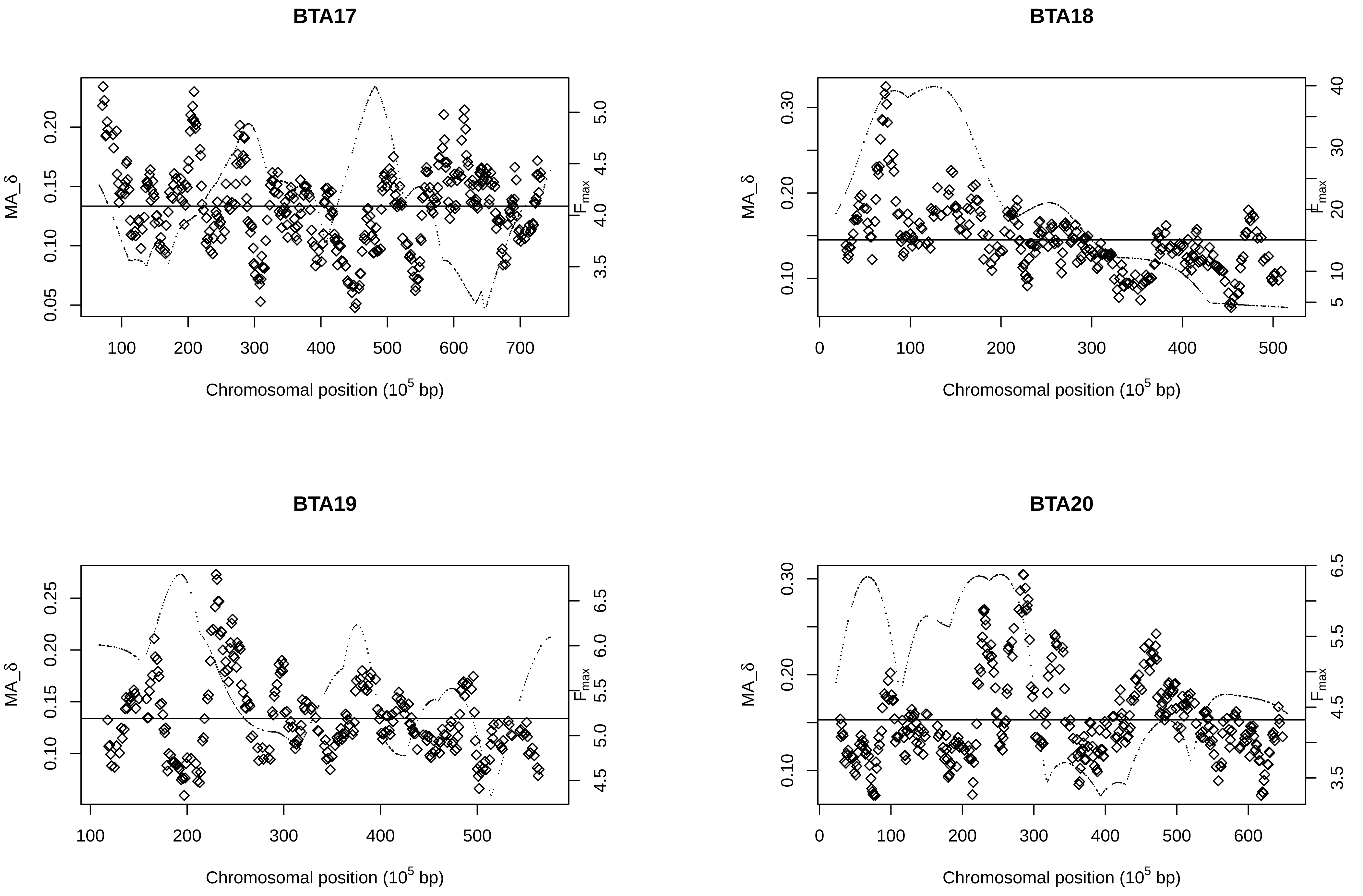
BTA17
BTA18
BTA19
BTA20
MA_δ
F_{max}
Chromosomal position (10^5 bp)

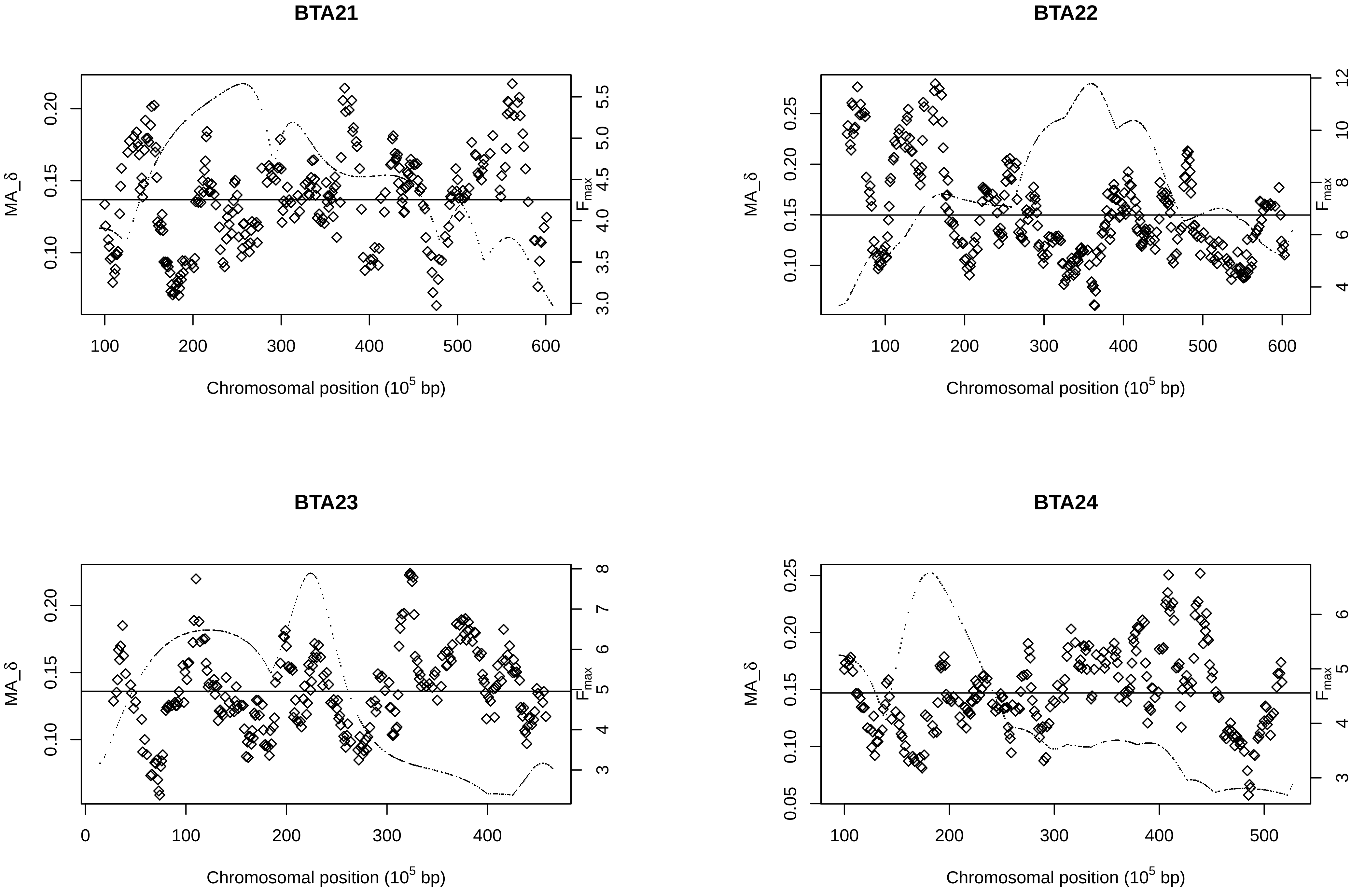

BTA21
BTA22
BTA23
BTA24
MA_δ
F_{max}
Chromosomal position (10^5 bp)

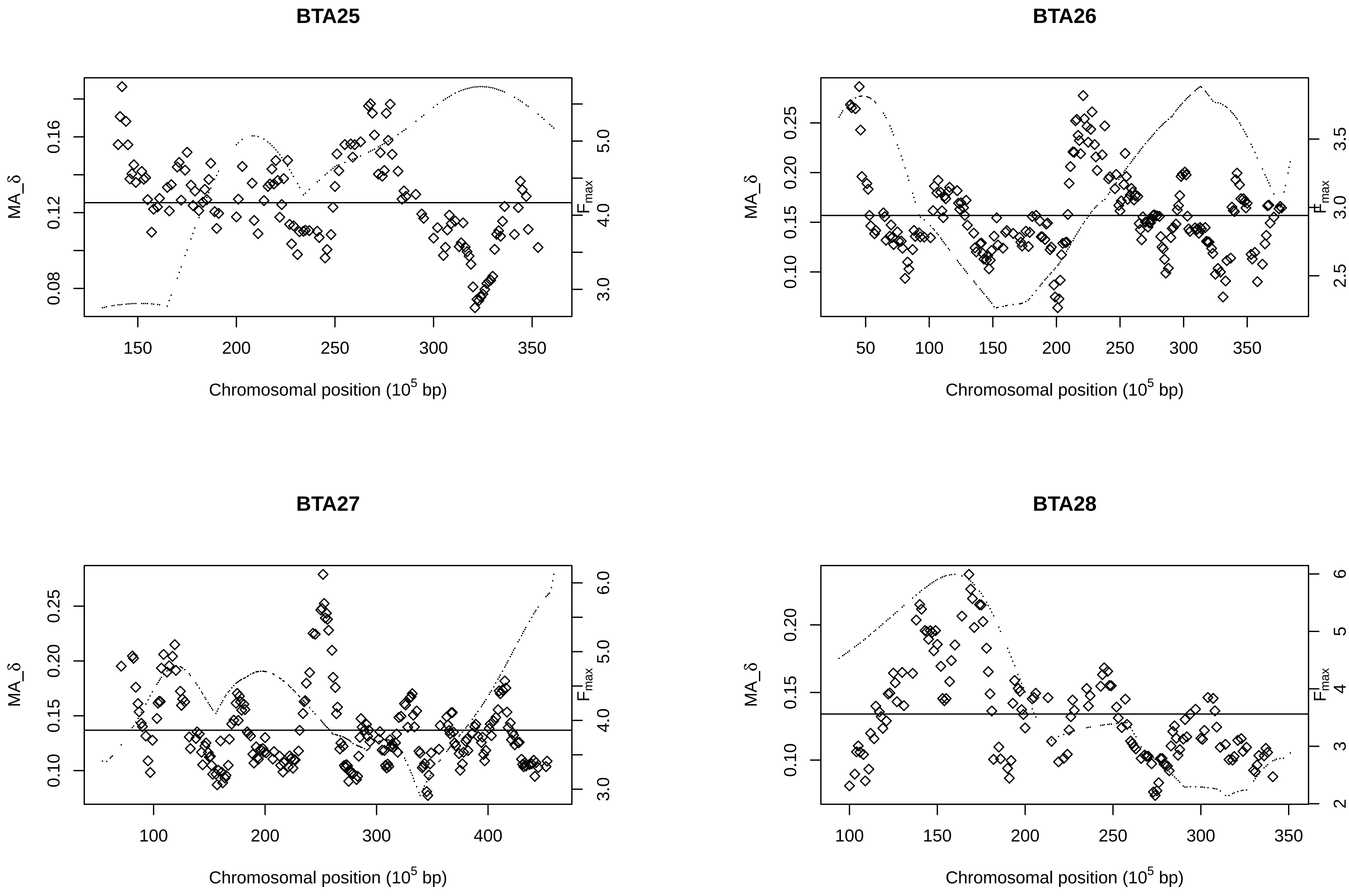
BTA25
MA_δ
F_max
Chromosomal position (10^5 bp)
BTA26
MA_δ
F_max
Chromosomal position (10^5 bp)
BTA27
MA_δ
F_max
Chromosomal position (10^5 bp)
BTA28
MA_δ
F_max
Chromosomal position (10^5 bp)

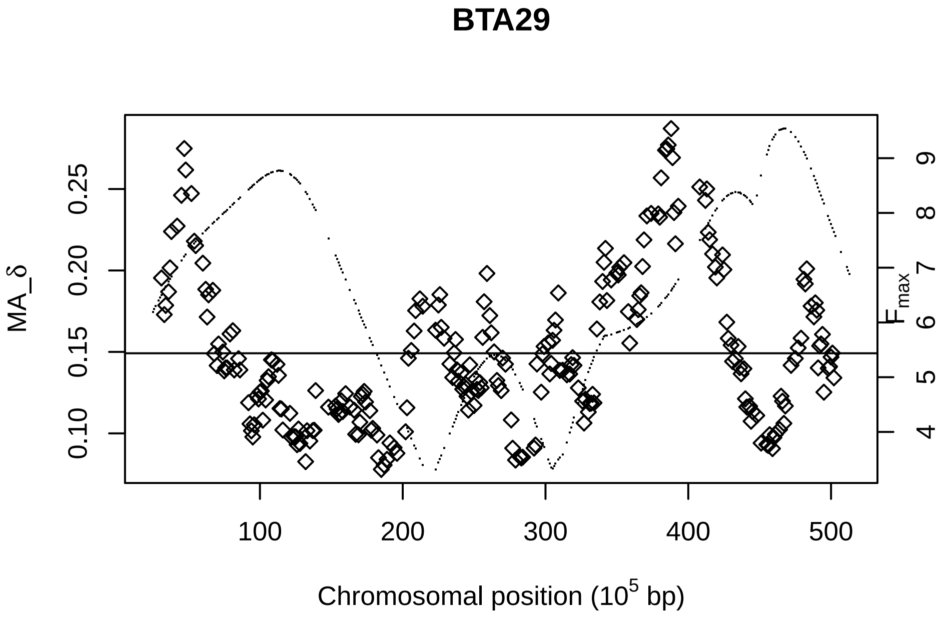

BTA29
MA_δ
0.10
0.15
0.20
0.25
F_{max}
4
5
6
7
8
9
100
200
300
400
500
Chromosomal position (10^5 bp)
