# Supplementary material for: Information content in genome-wide scans: concordance between patterns of genetic differentiation and linkage mapping associations
Source: BMC Genomics. 2011 Jan 26;12:65. doi: 10.1186/1471-2164-12-65 (PMC3041744; doi:10.1186/1471-2164-12-65)
Supplement: Additional file 3 — Table S2: Top 1% MA_δ values, sorted by chromosome. [file 1471-2164-12-65-S3.DOC]

Table S2. Top 1% MA_δ values, sorted by chromosome.

| chrom | position  (10^5^ bp) | MA_δ |
| --- | --- | --- |
| 1 | 1002 | 0.268021 |
| 2 | 931 | 0.269291 |
| 3 | 361 | 0.263072 |
| 3 | 362 | 0.272529 |
| 3 | 694 | 0.268835 |
| 3 | 697 | 0.282457 |
| 5 | 598 | 0.272547 |
| 5 | 599 | 0.289489 |
| 5 | 606 | 0.268269 |
| 5 | 610 | 0.322432 |
| 5 | 613 | 0.310373 |
| 5 | 614 | 0.291485 |
| 5 | 616 | 0.303514 |
| 5 | 617 | 0.310007 |
| 5 | 618 | 0.305684 |
| 5 | 621 | 0.298211 |
| 5 | 622 | 0.289688 |
| 5 | 625 | 0.305973 |
| 5 | 629 | 0.312318 |
| 5 | 630 | 0.277427 |
| 5 | 636 | 0.298956 |
| 5 | 639 | 0.321181 |
| 5 | 640 | 0.289229 |
| 5 | 645 | 0.28289 |
| 5 | 646 | 0.28021 |
| 5 | 647 | 0.278097 |
| 6 | 713 | 0.266229 |
| 6 | 728 | 0.271264 |
| 6 | 729 | 0.264341 |
| 6 | 730 | 0.263063 |
| 6 | 731 | 0.271752 |
| 6 | 766 | 0.266722 |
| 6 | 769 | 0.289387 |
| 6 | 770 | 0.286589 |
| 6 | 773 | 0.312913 |
| 6 | 774 | 0.324818 |
| 6 | 775 | 0.321211 |
| 6 | 776 | 0.321211 |
| 6 | 777 | 0.324715 |
| 6 | 778 | 0.293184 |
| 6 | 779 | 0.293104 |
| 7 | 413 | 0.272661 |
| 7 | 417 | 0.265008 |
| 7 | 421 | 0.273246 |
| 7 | 422 | 0.271648 |
| 7 | 534 | 0.268591 |
| 7 | 535 | 0.284744 |
| 7 | 537 | 0.27255 |
| 7 | 639 | 0.263774 |
| 8 | 460 | 0.262216 |
| 8 | 464 | 0.265706 |
| 8 | 473 | 0.263509 |
| 12 | 482 | 0.275642 |
| 12 | 483 | 0.295168 |
| 12 | 484 | 0.262156 |
| 13 | 111 | 0.267798 |
| 13 | 112 | 0.286849 |
| 13 | 113 | 0.304057 |
| 13 | 114 | 0.290738 |
| 13 | 115 | 0.272003 |
| 13 | 297 | 0.276226 |
| 13 | 431 | 0.275971 |
| 13 | 433 | 0.288689 |
| 13 | 434 | 0.279421 |
| 13 | 435 | 0.274622 |
| 13 | 436 | 0.284212 |
| 13 | 437 | 0.265517 |
| 13 | 438 | 0.268567 |
| 13 | 440 | 0.264165 |
| 13 | 441 | 0.288307 |
| 14 | 103 | 0.26758 |
| 14 | 104 | 0.284359 |
| 14 | 105 | 0.276757 |
| 14 | 106 | 0.289129 |
| 14 | 107 | 0.292985 |
| 14 | 108 | 0.28959 |
| 14 | 109 | 0.278783 |
| 14 | 110 | 0.282093 |
| 14 | 111 | 0.262742 |
| 16 | 664 | 0.276521 |
| 16 | 665 | 0.279605 |
| 16 | 666 | 0.264638 |
| 18 | 67 | 0.263075 |
| 18 | 69 | 0.286113 |
| 18 | 70 | 0.284744 |
| 18 | 72 | 0.31618 |
| 18 | 73 | 0.32458 |
| 18 | 74 | 0.304185 |
| 18 | 75 | 0.282617 |
| 19 | 230 | 0.272946 |
| 19 | 231 | 0.268179 |
| 20 | 229 | 0.267548 |
| 20 | 230 | 0.265515 |
| 20 | 231 | 0.267957 |
| 20 | 279 | 0.268174 |
| 20 | 281 | 0.287699 |
| 20 | 283 | 0.264901 |
| 20 | 285 | 0.304635 |
| 20 | 286 | 0.30412 |
| 20 | 288 | 0.290347 |
| 20 | 289 | 0.267414 |
| 20 | 290 | 0.267999 |
| 20 | 291 | 0.272199 |
| 20 | 292 | 0.278693 |
| 22 | 65 | 0.276379 |
| 22 | 162 | 0.272482 |
| 22 | 163 | 0.279558 |
| 22 | 168 | 0.274754 |
| 22 | 171 | 0.268441 |
| 26 | 38 | 0.268417 |
| 26 | 39 | 0.265332 |
| 26 | 42 | 0.264242 |
| 26 | 45 | 0.286522 |
| 26 | 221 | 0.277485 |
| 27 | 252 | 0.278968 |
| 29 | 47 | 0.274867 |
| 29 | 384 | 0.27382 |
| 29 | 385 | 0.274602 |
| 29 | 386 | 0.277052 |
| 29 | 388 | 0.287107 |
| 29 | 389 | 0.269267 |
